# Numerical and Structural Genomic Aberrations Are Reliably Detectable in Tissue Microarrays of Formalin-Fixed Paraffin-Embedded Tumor Samples by Fluorescence In-Situ Hybridization

**DOI:** 10.1371/journal.pone.0095047

**Published:** 2014-04-14

**Authors:** Heike Horn, Julia Bausinger, Annette M. Staiger, Maximilian Sohn, Christopher Schmelter, Kim Gruber, Claudia Kalla, M. Michaela Ott, Andreas Rosenwald, German Ott

**Affiliations:** 1 Department of Clinical Pathology, Robert-Bosch-Krankenhaus and Dr. Margarete Fischer-Bosch Institute of Clinical Pharmacology, Stuttgart, Germany; 2 Institute of Pathology, University of Würzburg, Würzburg, Germany; 3 Institute of Pathology, Caritas-Hospital, Bad Mergentheim, Germany; Istituto dei tumori Fondazione Pascale, Italy

## Abstract

Few data are available regarding the reliability of fluorescence in-situ hybridization (FISH), especially for chromosomal deletions, in high-throughput settings using tissue microarrays (TMAs). We performed a comprehensive FISH study for the detection of chromosomal translocations and deletions in formalin-fixed and paraffin-embedded (FFPE) tumor specimens arranged in TMA format. We analyzed 46 B-cell lymphoma (B-NHL) specimens with known karyotypes for translocations of *IGH*-, *BCL2-*, *BCL6*- and *MYC*-genes. Locus-specific DNA probes were used for the detection of deletions in chromosome bands 6q21 and 9p21 in 62 follicular lymphomas (FL) and six malignant mesothelioma (MM) samples, respectively. To test for aberrant signals generated by truncation of nuclei following sectioning of FFPE tissue samples, cell line dilutions with 9p21-deletions were embedded into paraffin blocks. The overall TMA hybridization efficiency was 94%. FISH results regarding translocations matched karyotyping data in 93%. As for chromosomal deletions, sectioning artefacts occurred in 17% to 25% of cells, suggesting that the proportion of cells showing deletions should exceed 25% to be reliably detectable. In conclusion, FISH represents a robust tool for the detection of structural as well as numerical aberrations in FFPE tissue samples in a TMA-based high-throughput setting, when rigorous cut-off values and appropriate controls are maintained, and, of note, was superior to quantitative PCR approaches.

## Introduction

A variety of methods are available for the detection of genetic aberrations in the routine diagnostic work-up of tumor specimens. Of those, classical chromosome banding and fluorescence in-situ hybridization (FISH) are of major importance. Although conventional cytogenetic approaches are still considered to be the gold standard in genetic diagnostics, allowing for the comprehensive analysis of tumor-specific alterations in the karyotype, banding analyses are limited by the availability of fresh material and by the need for in-vitro cultivation of tumor cells. In contrast, FISH analyses are not restricted to metaphase spreads prepared from freshly isolated dividing tumor cells, but can be performed on formalin-fixed paraffin-embedded (FFPE) interphase nuclei. Moreover, FISH can detect sub-clonal genetic changes, that may be missed by chromosome banding, therefore offering superior sensitivity in comparison with conventional cytogenetic approaches [1]. Of importance, a major advantage of FISH assays is the possibility to perform hybridizations in high-throughput approaches using tissue microarrays (TMA) in diagnostic settings, and especially for retrospective studies of archival pathological specimens. FISH on TMAs facilitate an expeditious analysis of specific genetic alterations in up to hundred tumor samples on a single slide, therefore enormously reducing costs and execution time, while keeping the experimental procedure constant [2]. A number of these high-throughput studies have been performed on tumor specimens and it is generally agreed that translocations and gene amplifications can be readily detected in FFPE TMA formats [3]–[5]. A meticulous analysis of the reliability of the results of translocation analyses on TMAs, however, has not been convincingly done so far, and it is still a matter of debate whether deletions can be reliably detected using the TMA approach.

In order to address these open questions, we investigated a panel of B-cell lymphoma (B-NHL) and malignant mesothelioma samples. Since two main settings have to be considered in the analysis of the reliability of FISH, rearrangement and copy number evaluation studies, especially the detection of deletions, we employed

1. a panel of FISH break-apart probes, detecting the hallmark lymphoma-associated translocations of the *IGH-, BCL2-, BCL6-* and *MYC*-loci in 46 B-NHL samples, respectively, the genetic aberrations of which had previously been determined by classical banding analysis and that served as internal control using the gold standard.
2. locus-specific DNA-probes in order to detect numerical aberrations, especially deletions, on FFPE TMA tumors of 62 B-NHL and six malignant mesothelioma samples.

Also pertaining to the latter point, we compared TMA-FISH to the hybridization of whole tissue sections, as well as to a quantitative real-time PCR (qPCR) approach targeting several genetic loci.

## Results

### Translocations of *BCL2*, *BCL6*, *MYC* and *IGH* can be reliably detected by FISH on TMAs

A total of 46 B-NHL tumor samples with available chromosome banding data had been spotted on TMAs. Following initial testing as described in the Materials and Methods section, all 46 tumor samples spotted on TMAs were hybridized with the four break-apart probes, thus performing a total of 184 hybridizations. A low hybridization quality without evaluable signals or loss of TMA scores following pretreatment was observed in 13/184 hybridizations (7%) (Table S1), and these cases were excluded from further analysis.

Taking into account only the chromosomal bands harboring BCL2-, BCL6-, IGH- and MYC loci, translocations had been detected in 32/46 tumor samples by chromosome banding, while FISH analysis revealed breaks in 30/46 samples (Table 1, Table S1). The mean frequency of cells with signal constellations indicative of a translocation was 53% (range: 37–86%), 45% (range: 19–80%), 54% (range: 19–95%) and 34% (range: 24–50%), for BCL2, BCL6, IGH and MYC, respectively. There were no significant differences in the frequency of cells showing breaks, when different lymphoma entities were separately analyzed. Similar numbers of cells showing translocation signals were seen in 51 B-NHL samples that were investigated by FISH on whole tissue sections in a routine diagnostic setting at the Department of Clinical Pathology, Robert-Bosch-Hospital, Stuttgart (data not shown). In this unrelated series, lymphomas with BCL2 or MYC translocations showed a minimum of 32% and 25% cells with signals indicative for rearrangements, respectively. Since the number of cells showing translocation signals reached 94% in MYC-positive samples, the mean frequency of cells with breaks in MYC rearranged samples was higher (67%, range: 25% to 94%) than the frequency of positive cells in BCL2-rearranged cases (53%, range: 32% to 75%). Not unexpectedly, the MYC-positive sample with a low translocation frequency of 25% cells with breaks was a (“double-hit”) FL, while the remaining samples with 40% to 94% positive cells were all DLBCL and Burkitt lymphomas (BL), obviously reflecting different numbers of non-tumorous bystander cells in the respective entities.

**Table 1:** Overview on the results obtained in lymphoma and MM specimens analyzed for translocations and genomic deletions, performing chromosome banding (CB), FISH on TMA format or on whole tissue sections (WTS), and qPCR.

	Chromosome Banding	TMA FISH	WTS FISH	qPCR	Concordance* CB-FISH	Concordance* TMA-WTS FISH	Concordance* FISH-qPCR
**Structural genomic aberrations**							
- BCL2-Translocations (Chr. 18q21)	5/46	6/46	5/25	-	97.8%	100%	-
- BCL6-Translocations (Chr. 3q27)	6/46	11/46	10/25	-	86.3%	100%	-
- MYC-Transocations (Chr. 8q24)	3/46	4/46	2/25	-	94.9%	100%	-
- IGH-Translocations (Chr. 14q32)	18/46	20/46	13/25	-	95.3%	100%	-
**Numerical genomic aberrations**							
With Chromosome Banding Data	-						
- ATG5-Deletions (Chr. 6q21)	10/62	10/62	10/62	2/10	100%	100%	20%
- Normal ATG5 (Chr. 6q21)	20/46	20/46	20/46	20/46	100%	100%	100%
Without Chromosome Banding Data							
- ATG5-Deletions (Chr. 6q21)	-	16/62	-	11/16	-	-	68.8%
- CDKN2A-Deletions (Chr. 9p21)	-	6/6	-	4/6	-	-	66.7%

Also, the concordance levels obtained for the respective investigations (* concordance level of analyzable specimens) are given.

Translocations involving *BCL2* on chromosome 18q21 were observed in five lymphomas by chromosome banding, one of them with an unbalanced translocation without identifiable partner. FISH analysis with the *BCL2* break-apart probe disclosed breaks at 18q21 in all these cases and, additionally, in one tumor sample without obvious translocation in the karyotype (Table S1). More complex karyotypic alterations were observed for the *BCL6* gene locus in classical cytogenetics including inversions (n = 1), insertions (n = 1), duplications (n = 2) and deletions (n = 2) affecting chromosomal band 3q27. While the samples with insertions and duplications of 3q27 showed clear split signals using the *BCL6* break-apart probe, the three deletions and inversions remained undetected with FISH analysis. Translocations affecting 3q27 were revealed in six tumor samples by karyotyping (two of them with unidentified translocation partners). In all cases, FISH on TMAs disclosed these translocations. In addition, FISH revealed chromosomal breaks involving the *BCL6* gene in five B-NHL patients without aberrant chromosome 3q27 region by banding (Table S1). Translocations involving 8q24 harboring the *MYC* gene locus were observed in three cases by karyotyping, and in all tumors a *MYC*-break was evident also upon hybridization with the *MYC* break-apart probe. Moreover, one more B-NHL sample was found to be translocation-positive in the FISH approach, while this translocation remained undetected by chromosome banding (Table S1). Translocations affecting 14q32, involving the *IGH* locus, were observed in 18 B-NHL on the karyotypic level. FISH results demonstrated corresponding results for *IGH*-breaks in all samples. In addition, two more cases showed translocations of the *IGH*-gene locus by FISH, but not by banding (Table S1). The number of translocations detected for *BCL2* and *MYC*, of note, paralleled those encountered for *IGH*, thus indicating that the main rearrangements of these oncogenes associated with the *IGH* gene locus were covered with this approach (9/10, 90%). However, some genetic aberrations occurred, especially involving the *BCL6* gene (9/17, 53%), that were not associated with a juxtaposition to the *IGH* gene locus, reflecting the aforementioned deletions, duplications, insertions and inversions that affected *BCL6*.

In conclusion, a total of 171/184 hybridizations (93%) yielded adequate hybridization quality, enabling the accurate FISH-based evaluation of translocation signals (Table 1, Table S1). There were only slight differences in the evaluation of the cases by the two independent observers. Specifically, 8/171 hybridizations (5%), in which the number of cells with break-apart signals were close to the cut-off ratio determined, were evaluated positive by observer 1 and negative by observer 2. Re-hybridization of these samples in whole tissue sections then resulted in concordant evaluation by the two observers (all evaluated as negative). Comparing results obtained by chromosome banding and FISH, concordant findings were observed in 159/171 calls (93%) (Table S1). The number of cases with genetic alterations detected solely by FISH was 9/171 (5%). To avoid the generation of false-positive results obtained by FISH on TMA format, these discrepant samples were subsequently re-hybridized on whole tissue sections. In 7/9 tissue samples the results initially obtained by TMA-FISH could be validated also on whole tissue sections, while the remaining two specimens failed to yield analyzable results in that format (Table 1, Table S1). In 3/171 cases (2%), aberrations (deletions and inversions of *BCL6*) were observed only by classical cytogenetics.

In addition, FISH analysis was repeated in 25 of 46 samples initially studied on TMAs using whole tissue sections by re-hybridizing them with break-apart probes for *BCL2*, *BCL6*, *MYC* and *IGH*. Of these 100 hybridization events, 12 (12%) were not evaluable on the TMAs due to inadequate pepsin digestion or loss of tissue scores following pretreatment. All 88 evaluable hybridizations (hybridization efficiency: 88%) showed concordant results in 100% on the TMA and the whole tissue sections (Table 1, Table S1).

### A tumor cell poulation exceeding 25% with deletions is required to reliably detect genetic deletions in tissue sections

**Figure 1:**
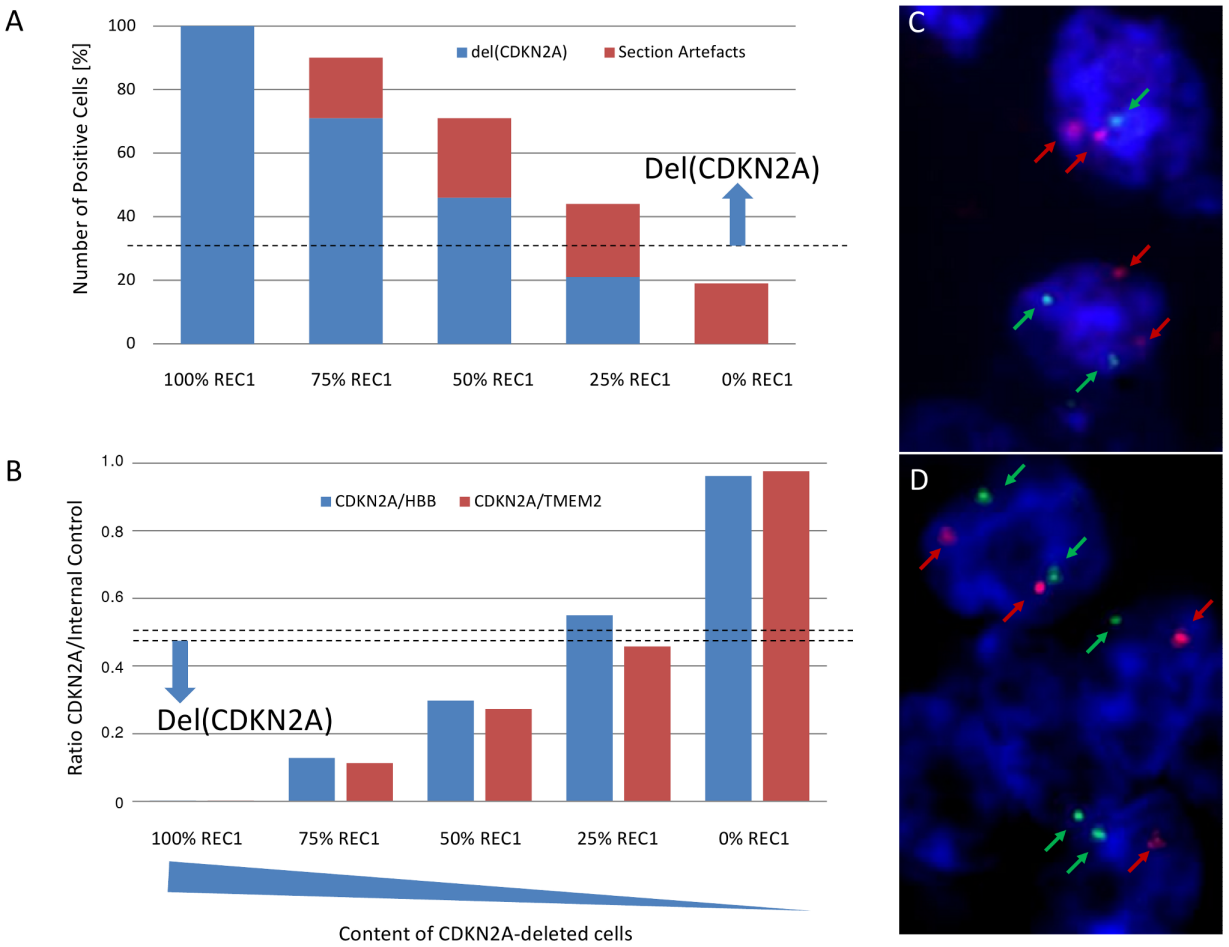
Experimental set up for the accurate testing of numerical genetic alterations in FFPE tumor tissue performing FISH (A) or qPCR (B). Using JVM2 and REC1 MCL cell lines, cell dilutions containing different amounts of *CDKN2A*-deleted cells were established (ranging from 0% to 100% *CDKN2A*-deleted cells). Representative FISH results (C–D), indicating the occurrence of section artefacts in cell suspensions containing only non-deleted *CDKN2A* (100% JVM2), by obtaining aberrant signal constellations not matching the expected signal distribution of two red and two green copies (*CDKN2A*: marked in red; 9cen: marked in green, magnification: 100×). The cut-off level obtained from investigation of reactive lymph node specimens is depicted as dashed line for the FISH (A) and the qPCR (B) analysis.

To obtain insight into the accurate FISH-based detection of genetic deletions in tissue sections, FFPE blocks were created from MCL cell line suspensions with a defined tumor cell content. Hybridization with the *CDKN2A*-specific probe resulted in the detection of two signals in >70% nuclei in the cell block containing pure JVM2 cells. In contrast, pure REC1 cells showed homozygous deletion of the *CDKN2A* gene, while maintaining four copies of the centromeric region (polyploid cells, DSMZ). In an experimental setting, these cell lines were mixed at defined levels, thus reducing the number of cells deleted for *CDKN2* successively (Figure 1A and C–D). The number of truncated cells created by sectioning of the FFPE block, therefore, could be estimated: The section artefact was defined as the difference between the total number of cells with reduced *CDKN2A* signals and the expected number of those nuclei. As shown in Figure 1A and C–D, the number of section artefacts varied between 17% and 25%. Therefore, equally taking into account the mathematical *CDKN2A* cut-off ratio for deletion of 30%, as calculated from FISH analysis of the negative controls, the number of *CDKN2A*-deleted cells should exceed 30%, to reliably detect the genetic alteration (Figure 1A).

Similar results were obtained using a qPCR approach. Wild type *CDKN2A* with both copies maintained was observed in 100% of JVM2 cells, while no gene copy was detected in REC1 cells. With increasing numbers of REC1 cells in the REC1-JVM2 dilutions, a linear reduction of the *CDKN2A*-signal was obvious. Reactive lymph node samples showed *CDKN2A*/*HBB* and *CDKN2A*/*TMEM2* ratios ranging from 0.63 to 1.06, thus defining cut-off values for *CDKN2A*-deletions of 0.52 and 0.54, respectively (Figure 1B). Similar to the FISH assay, a proportion of *CDKN2A*-deleted cells higher than 25% was required for a reliable detection of deletions (Figure 1B).

### FISH analysis has a superior sensitivity in the detection of numerical genetic alterations in FFPE tumor samples in comparison with qPCR

To clarify whether FISH or qPCR is the more sensitive approach for an adequate detection of selected deletion regions in FFPE tissues, we used six malignant mesothelioma (MM) FFPE samples, since MM have been shown to harbor *CDKN2A*-deletions in 25–50% of cases [6], [7]. In keeping with these data, two cases turned out to harbor monoallelic *CDKN2A* deletions by FISH (in 36%–47% of cells), while in four of them a loss of both alleles was disclosed (in 32–75% of cells). Using the qPCR approach in those cases with *HBB* and *MAL* as internal controls, the deletions detected by FISH could be validated in all four samples with loss of both gene copies when referring to *HBB*, and in one of them when referring to *MAL*. Monoallelic *CDKN2A* deletion was not detectable by qPCR (Table 1, Table S1).

**Figure 2:**
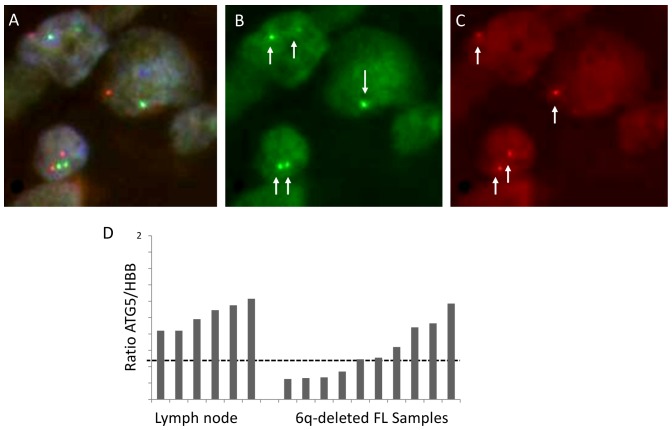
Comparison of FISH and qPCR in FL samples. Ten samples with detectable 6q-deletions as observed in chromosome banding (positive controls) were investigated by FISH (A–C) and by qPCR (D). FISH was performed with 6q21- (red) (ATG5) and 6cen-specific (green) BAC-clones. Representative overlay of Dapi-, SpectrumOrange (red: R) and SpectrumGreen (green: G) filters (A), either single SpectrumGreen (B) or SpectrumOrange (C) filter indicating different signal constellations (wildtype: 2R2G; 6q21-deletion: 1R2G; section artefact: 1R1G) (magnification: 100×). Using the qPCR approach ten positive control samples were compared to five reactive lymph node specimens (D) (here referred to *HBB* as internal control). For the qPCR approach, the cut-level obtained is indicated as dashed line. Although these samples clearly harboured deletions in the chromosomal band 6q21 (including the *ATG5*-locus) as observed by FISH and chromosome banding, in only four of ten 6q-deleted FL samples loss of the *ATG5*-gene was detected by qPCR.

Ten (10/62) FL samples harboring deletions of 6q21 as detected by chromosome banding analysis, as well as 51 of 62 FL samples without cytogenetic data available were then investigated by FISH and qPCR (Table 1, Table S1). One sample was not evaluable by either method. Monoallelic deletion of *ATG5* in 6q21 was verified by FISH, both in TMA format and on whole tissue sections, using locus-specific BAC clones in the ten samples with positive cytogenetics (Table 1, Table S1). In these experiments, the mean number of affected cells was 48% (range: 30% to 70%) (Figures 2A–C). ATG5 deletions were also observed in 16/51 FL samples without cytogenetic data (50% of cells, range: 34 to 80%). In the 35 remaining samples, signal constellations indicative of deletions were observed in numbers suggesting sectioning artefacts (15%–28%). All 26 tumor samples with deletions of the ATG5 locus as verified by FISH and the remaining 35 samples with deletion signals below the cut-off level were then analyzed by qPCR. Of the 26 cases with proven 6q deletion by FISH, only 13 samples (with 65% to 80% deletion-positive cells) showed an ATG5 deletion by qPCR (Table 1, Table S1). Seven samples with 30% to 60% deletion-positive cells by FISH, showed ATG5/HBB and ATG5/TMEM2 ratios slightly exceeding the cut-off level of 0.46 and 0.47 for ATG5/HBB and ATG5/TMEM2, respectively (ranging from 0.49 to 0.64). In the six remaining cases exhibiting clear-cut deletions by FISH, no constellation indicative of a gene deletion (Figure 2C–D) was seen.

In addition, 20 FL samples from the initial test set of 46 B-NHL without genomic aberrations of the 6q21-chromosomal region as shown by chromosome banding were tested by FISH using DNA probes directed against 6q21, and by PCR. None of the samples investigated harbored deletions of the *ATG5* gene by FISH neither on TMA nor on whole tissue sections (showing only sectioning artefacts with signal constellations indicative of deletions not exceeding 29%). qPCR failed to reveal deletions as well (Table 1, Table S1).

In conclusion, reliable results obtained by FISH analysis could not be reproducibly validated by qPCR, either because of an inadequate content of tumor cells showing deletions in the specimens, or – in theory - due to underlying genetic aberrations affecting the reference gene loci and thus the appropriate evaluation of qPCR results in the tumor samples.

## Discussion

Numerous studies have been conducted using DNA probes for the detection of lymphoma-associated primary translocations. In the majority of these studies, FISH turned out to be more sensitive in comparison with conventional karyotyping [8]. Few data, however, are available concerning the reliability of FISH in a high-throughput setting using TMAs in solid tumors and hematological malignancies [3]–[5]. In these studies, however, the technical feasibility and the robustness of the TMA hybridization method were mainly evaluated. Moreover, these studies used different DNA probes for either amplifications or translocations on various tumor entities without adjusting the results to data available from alternative cytogenetic approaches. Therefore, it was of considerable interest to us to perform a comprehensive study in order to meticulously assess the reliability of FISH analysis in a high-throughput TMA setting not only for the widely used break-apart probes, but especially also for the discovery of deletions in FFPE tissue samples. Thus, we analyzed 46 B-NHL samples with known karyotypes spotted on TMAs for hallmark lymphoma-associated translocations of the *IGH*-, *BCL2*, *BCL6*- and *MYC*-genes. FISH analysis was performed with a panel of commercially available break-apart probes, and results were compared also with data obtained from whole tissue sections of corresponding samples. In summary, a total of 171/184 hybridizations (94%) on the TMA format had appropriate hybridization quality, enabling an accurate evaluation of FISH signals. This result clearly indicates the excellent technical prerequisites in our study, since comparable results had previously been published, reporting a hybridization efficiency of more than 80% [3]–[5], [9]. Of those 171 hybridizations, 159 results (93%) were matching data from classical chromosome banding. Of note, the FISH approach was highly sensitive in the detection of translocations of *BCL2*, *BCL6, MYC* and *IGH*, as well as of duplications and insertions involving *BCL6* within this study cohort. In addition, the combined banding/interphase cytogenetic approach revealed 9/171 (5%) alterations which were detected *solely* by FISH. Considering these data, FISH analysis emerged as a very powerful tool for the detection of translocations, in some conditions exceeding the sensitivity of the chromosome banding approach, in keeping with previous studies [10], [11]. This of course holds especially true in samples with a low or no yield of clonally aberrant metaphases in the conventional cytogenetic workup, as shown also in 5 of 9 tumor samples in our series (2 to 4 metaphases analyzed, with only 2 to 3 clonal metaphases). In four cases translocations were detected only by FISH. Similar data have been emerged from previous studies indicating clonal heterogeneity and the fact that selected tumor subclones might overgrow others *in vitro*, thus resulting in an underestimation of subpopulations not detectable with conventional banding approaches [11], [12]. On the other hand, in our study, chromosome banding turned out to be superior to FISH analysis with regard to the detection of inversions and deletions involving band 3q27/the *BCL6* gene locus, which obviously reflects the specificity of the *BCL6* break-apart probe to detect numerous translocations/aberrations in the alternate breakpoint region (ABR) of the *BCL6* gene, but not aberrations occurring outside this region. Conventional chromosome banding allows for a genome-wide screening of tumor-specific cytogenetic aberrations within the order of resolution of the karyotype. FISH analysis, in contrast, represents a highly sensitive and time-effective alternative for the detection of targeted genetic alterations. Our work especially points out the fact that targeted FISH is reliably feasible in FFPE tissue TMA samples: results from TMA hybridizations were fully reproducible when compared to whole tissue section FISH of randomly assigned samples and in tumor specimens showing discordant results of TMA-FISH and chromosome banding. In a recent large study, in which cases from different institutions had been assembled, hybridization efficiencies ranging from 87% to 92% in the different TMAs were achieved, thus proving the robustness of the assay. Moreover, highly satisfactory kappa values of 0.92 to 0.94 were obtained in that study when FISH results were evaluated between independent observers in different institutions [9]. Finally, a round robin test recently conducted in Germany proved the high reproducibility of FISH evaluation among different institutions [13]. These studies, therefore, provide evidence for a high concordance rate of classical cytogenetics and hybridizations of TMAs and whole tissue sections. Only 13% of samples were not evaluable on the TMA format due to inadequate pepsin digestion or loss of tissue cores following pretreatment, and thus missing complete coverage of adequate hybridization terms in all the different TMA-samples. A dependence of hybridization efficiency also on the lymphoma subtype has been recently shown [5].

While FISH analysis, in conclusion, clearly produces robust results for the assessment of lymphoma-associated translocations, the suitability of the FISH approach for the detection of deletions of genetic material in FFPE tissue samples still is controversially discussed [1], [14]. In fact, some pitfalls do exist when searching for copy number alterations in routine worked-up FFPE tissues. In lymphomas, deletions are often representing secondary aberrations that may not be present in the entire tumor cell population, but only in tumor subclones. Moreover, the signal constellation identified may often reflect the distribution of signals obtained from truncated nuclei, when a part of the nucleus is lost following sectioning of slides. An accurate establishment of the cut-off level for the assessment of deletions, therefore, is highly recommended, with adequate definition of negative controls [1], [14]. In order to address this question, we established an experimental set-up allowing for the exact definition of the number of cells harboring deletions in FFPE tissue, and performed a rigorous analysis comparing FISH in TMA-format and on whole tissue sections with both chromosome banding and qPCR in order to identify the best-suited approach for the detection of numeric alterations in FFPE tumor samples.

When analyzing serially diluted cell line samples containing exactly defined numbers of cells with a given deletion, we were able to determine a crucial threshold at a minimum of 25% of cells containing the deletion, since 17% to 25% of cells turned out to harbor truncated nuclei in every sample analyzed. In our study, considering the number of up to 25% truncated nuclei identified in an experimental setting on the one hand, and the calculated cut-off ratio of 30% on the other hand, only samples harboring deletion-like signal constellations in ≥30% of cells were designated as positive. Similar high cut-off ratios ranging between 20% to 41% have also been proposed in previous publications [14], [15]. These studies, however, had been performed on whole tissue sections only. Thus, to the best of our knowledge, the present study is the first to assess the robustness of detecting deletions in FFPE tissue samples spotted on TMAs. Losses of genetic material detected by FISH analysis in the present study occurred in 32% to 75% of cells in mesothelioma samples (mean: 52%) and in 38% to 70% of cells in FL samples (mean: 52%). The large range of results in FL most probably reflects the different composition of the cellular microenvironment in these lymphomas, with largely varying numbers of non-tumorous bystander cells in different samples. Interestingly, qPCR could validate the FISH results only in samples with biallelic deletions and in specimens with monoallelic deletions, but a high tumor cell content, although the qPCR approach had been successfully established in an experimental model set up. There are several explanations to explain the inferiority of qPCR when compared with FISH in the FFPE tumor samples. Successful PCR analysis requires DNA of adequate quality, which is often limited when working-up FFPE specimens [16]. Moreover, when investigating tumor samples, underlying genetic alterations generally have to be considered, possibly also affecting the loci of reference genes used. In our study, for example, we could not exclude that reference loci harbored copy number changes and thus, influenced the measurement of the target genetic region. This caveat obviously turned up in three MM tumor sample, that was deletion-positive when referred to *HBB*, but not to *MAL* as internal control.

In conclusion, FISH represented an excellent tool for the detection of structural as well as numerical aberrations in FFPE tissue samples in a TMA-based high-throughput setting in an experimental study. It should be kept in mind, however, that secondary genetic lesions might not be representative for the whole tumor population. Therefore, the application of rigorous cut-off values and the usage of appropriate controls is required. When carefully considering these aspects, FISH is a highly reliable and reproducible method allowing for the rapid detection of deletions on TMAs the sensitivity of which obviously exceeds that of a qPCR approach.

## Materials and Methods

### Tumor Specimens

Archival FFPE tissue blocks of 46 B-NHL (all with chromosome banding data available) were punched and arranged on a TMA format. Of these specimens, 24 were primary nodal diffuse large B-cell lymphomas (DLBCL), 11 extranodal DLBCL, and 11 follicular lymphomas (FL). Whole tissue sections for FISH analysis were cut from 25 of these cases. Numerical genetic alterations were analyzed in 62 follicular lymphoma (FL) (10 of 62 with cytogenetic data) and six mesothelioma specimens (without cytogenetic data) that had also been spotted on TMAs. All paraffin blocks were retrieved from the files of the Institute of Pathology, University of Würzburg, Germany and from the Department of Clinical Pathology, Robert-Bosch-Krankenhaus, Stuttgart, Germany. Chromosome banding analyses had been generated as a part of the diagnostic work-up for the 46 B-NHL tumor samples from 1993 to 2007 at the Institute of Pathology, University of Würzburg, Germany. All cases were reviewed and classified according to the criteria of the current World Health Organization (WHO) classification systems [17], [18]. The study was approved by the local Ethics committees, that also waived the need for written informed consent from the donor (Ethics committee of the Medical Faculty, Eberhard-Karls-University and University Hospital Tübingen and Ethics committee of the Medical Faculty, University of Würzburg), in accordance with the Declaration of Helsinki.

### Cell Lines

To obtain insight into the reliability of testing numerical genetic alterations (especially deletions) in FFPE tissues, mantle cell lymphoma cell lines REC1 and JVM2 (DSMZ, Braunschweig, Germany) were utilized. These cell lines are well-characterized on the genetic level [19]. With special regard to the 9p21 locus, REC1 was shown to harbor a homozygous deletion of *CDKN2A*, while JVM2 retains two copies of *CDKN2A* [19]. Cell lines were cultured in RPMI-1640 medium (Life Technologies, Darmstadt, Germany), supplemented with 10% heat inactivated fetal bovine serum, 50 µg/ml streptomycin and penicillin (Life Technologies) and maintained in a humidified 95% air – 5% CO_2_ atmosphere at 37°C. In an experimental setting, REC1 cells were serially spiked into JVM2 cell suspensions to obtain suspensions with a defined content of *CDKN2A*-deleted cells (0%, 25%, 50%, 75%, 100% cells with deletion of *CDKN2A*). For comparison with the routine setting, 4×10^6^ cells of the cellular suspensions were washed with 100% isopropanol, fixed with buffered formalin and embedded into paraffin. Subsequent FISH analyses were performed on whole tissue sections.

### TMA construction

Tumor areas of paraffin tissue blocks were screened paying attention to the content of viable, well-fixed tissue. Three 0.6 mm thick cores were taken from the selected regions of the donor block with a ‘manual tissue puncher’ (Beecher Instruments, Silver Spring, Maryland, USA) and inserted into the recipient block. A total of four TMAs were constructed containing the 46 B-NHL (two TMAs with 20 and 26 samples, respectively) and the 62 FL together with six MM tumor samples (two TMAs with 34 samples, respectively).

### Chromosome banding

Metaphases had been prepared from short-term cell cultures of the 46 B-NHL cases according to standard protocols [20]. After classical Giemsa-trypsin banding, karyotypes were constructed according to the International System for Human Cytogenetic Nomenclature (ISCN). Identical structural aberrations, or genetic gains, in two or more metaphases and identical genetic losses in at least three metaphases were defined as clonal aberrations.

### FISH

Interphase-FISH was performed on 4 µm thick tissue sections. Hybridization was performed as previously described [9]. Briefly, whole tissue and TMA sections were deparaffinized, re-hydrated in 100, 80 and 70% ethanol, followed by incubation in 50 mM Tris-EDTA (pH 8.0) for 10 min at 100°C. The slides were then treated with pepsin (62.5 µg/ml in 0.01 N HCl) at varying times at 37°C, followed by dehydration in 70, 80 and 100% ethanol and denaturation for 10 min at 80°C. The slides were hybridized with 125 ng SO- and SG-labeled BAC-DNA probes, respectively, or following the manufactureŕs protocol for Vysis *BCL2*-, Vysis *IGH*- and Vysis *MYC* Break Apart FISH Probe Kits, and Vysis LSI *BCL6* (ABR) Dual Color, Break Apart Rearrangement Probe (Abbott Molecular, Wiesbaden-Delkenheim, Germany). After overnight hybridization at 37°C in a humid chamber, slides were washed and counterstained with 1.5 µg/ml DAPI in mounting medium (Axxora, Lörrach, Germany). In an initial test set, a TMA section covering 20 FL was treated with pepsin with varying incubation times (4 to 10 minutes) and hybridized with break-apart probes for the *BCL2*- and the *IGH*-gene loci (40 hybridization events). Hybridization efficiency was highest with 6 min pepsin digestion time yielding 80% to 94% samples with good hybridization results. This pretreatment condition was then applied to all subsequent FISH analyses. For the detection of chromosomal deletions involving *CDKN2A* in chromosome 9p21 the Vysis *CDKN2A/CEP* 9 FISH Probe Kit (Abbott Molecular) was used. Chromosomal deletions affecting 6q21 were analyzed applying the locus-specific bacterial artificial chromosome (BAC) probe RP11-106L23 (*ATG5*) (NCBI-ID: 206657, NC_000006.11, http://www.ncbi.nlm.nih.gov/clone/), in co-analysis with the chromosome 6 centromeric probe RP11-79O24 (NCBI-ID: 255610, NC_000006.11) (Source BioScience LifeSciences, Berlin, Germany), as previously described [21].

Tissue sections from three FFPE reactive lymph nodes had been used to determine the cut-off level for each probe. The reference range (cut-off) was defined as the mean copy number in lymph nodes plus three standard deviations. The cut-off levels were 10% (95% CI: 6.9–9.8), 8% (95% CI: 4.9–7.8), 9% (95% CI: 6.2–9.1) and 9% (95% CI: 5.9–8.8) for rearrangements of the *BCL2*-, *BCL6*, *IGH*- and *MYC* gene probes, respectively. For the deletion probes, the cut-off levels were set at 30% each for *CDKN2A* (calculated cut-off: 29%; 95% CI: 13.1–28.2) and *ATG5* (calculated cut-off: 28%; 95% CI: 12.1–27.2), respectively. Sections from three FFPE FL samples with known deletions in 6q by chromosome banding were used as positive controls. At least 100 intact nuclei per case were evaluated using an epifluorescence microscope (Leica Microsystems, Bensheim, Germany). Images were captured using the ISIS imaging system (MetaSystems, Altlussheim, Germany). For accurate evaluation of the results, two independent observers scored the signal constellation in 100 nuclei of two cores. The third core, as a rule, was evaluated only qualitatively to ensure equal signal distribution in all cores of one sample. In none of the samples discordant evaluation results were obtained for the three individual cores of one B-NHL case.

### Real-time quantitative PCR

To detect genomic deletions quantitative real-time PCR (qPCR) was performed as previously described [22]. Genomic DNA was extracted from 10 µm FFPE sections using the QIAamp DNA FFPE Tissue Kit (Qiagen, Hilden, Germany) according to the manufactureŕs protocol.

Primers for qPCR targeting *CDKN2A* (9p21) and *ATG5* (6q21) were designed based on published sequence data (NG_007485.1, NC_000006): CDKN2A_sense 5′-GATCCTTGTTAGCATTTCAG-3′; CDKN2A_antisense 5′-AGAGTGGAGGACCCGTG-3′; ATG5_sense 5′-AAGGGCCAACTGTGTACGTC-3′; ATG5_antisense 5′-CCATCTCTTGGCATTCCCTA-3′. As reference loci, *HBB* (11p15), *TMEM2* (9q21) and *MAL* (2q11) were selected based on the results of SNP-array and comparative genomic hybridization studies that had shown only infrequent genetic aberrations in these regions in B-NHL as well as in malignant mesothelioma [6], [23]. Previously described primers for *HBB* were applied [21]. Primers for *TMEM2* and *MAL* amplification were TMEM2_sense 5′-GCTTACAGGGTGGTGTTC-3′; TMEM2_antisense 5′-GGATCAGGATGTAATGAGTC-3′; MAL_sense 5′-TAATTGGAGCCCACGGTG-3′; MAL_antisense 5′-AGCGGTGCAGTGGTAGG-3′. Loss of chromosomal material was calculated by normalizing *ATG5* and *CDKN2A* qPCR data to the reference loci. The cut-off value for deletion was defined as the mean value in four to six reactive lymph nodes (FFPE) minus three standard deviations. The cut-off ratios for *ATG5/TMEM2 and ATG5/HBB* were 0.47 and 0.46, respectively. For *CDKN2A/TMEM2*, *CDKN2A/HBB and CDKN2A/MAL* cut-off ratios of 0.54, 0.52 and 0.50 were calculated, respectively. Genomic DNA extracted from three FL FFPE samples that showed deletions of the 6q-region by chromosome banding were used as positive controls. Genomic DNA of REC1 cells, that harbor a homozygous deletion of *CDKN2A* was used as positive control for the detection of losses in 9p21.

## Supporting Information

Table S1 **Detailed results of all experiments performed within the entire study cohort consisting of DLBCL, FL and MM specimens.** The numbers of cases studied and the individual in samples with chromosome banding data available, the FISH-probes used for the detection of either structural or numerical genomic aberrations, the number of cases investigated by either TMA-FISH or FISH of whole tissue sections and the number of cases additionally analyzed by qPCR are given. FISH results are provided as numbers of positive cells in [%], if aberrant, while qPCR results indicating genetic deletion are provided as ‘loss’. Abbrevations: DLBCL: Diffuse large B-cell lymphoma; FL: Follicular lymphoma; BAP: Break-apart probe; LSI: locus-specific identifier; Chr.: chromosome; WTS: whole tissue section; qPCR: quantitative real-time PCR on the DNA-level; Karyotypic abbreviations: der: derivative; del: deleted; bidel: biallelic deletion; dup: duplicated; add: added.
(XLSX)
